# Variability of Metal Levels in Spot, First Morning, and 24-Hour Urine Samples over a 3-Month Period in Healthy Adult Chinese Men

**DOI:** 10.1289/ehp.1409551

**Published:** 2015-09-15

**Authors:** Yi-Xin Wang, Wei Feng, Qiang Zeng, Yang Sun, Peng Wang, Ling You, Pan Yang, Zhen Huang, Song-Lin Yu, Wen-Qing Lu

**Affiliations:** 1 Department of Occupational and Environmental Health, School of Public Health, Tongji Medical College, Huazhong University of Science and Technology, Wuhan, Hubei, PR China; 2 Key Laboratory of Environment and Health, Ministry of Education & Ministry of Environmental Protection, and State Key Laboratory of Environmental Health (incubating), School of Public Health, Tongji Medical College, Huazhong University of Science and Technology, Wuhan, Hubei, PR China; 3 Department of Epidemiology and Biostatistics, School of Public Health, Tongji Medical College, Huazhong University of Science and Technology, Wuhan, China

## Abstract

**Background:**

Metals in single spot urine samples are often used to estimate individual exposure in human studies. However, measurements in urine could vary greatly over time due to variable exposure, potentially leading to exposure misclassification.

**Objective:**

We examined the variability of metal levels in the urine of 11 men who provided 529 samples on 8 days during a 3-month period, which corresponds to the duration of spermatogenesis.

**Method:**

The urinary levels of arsenic (As), cadmium (Cd), cobalt (Co), copper (Cu), lead (Pb), molybdenum (Mo), and nickel (Ni) were measured using inductively coupled plasma–mass spectrometry. We calculated the intraclass correlation coefficients (ICCs) to assess the reproducibility of metal measures and computed the sensitivity and specificity to evaluate how well spot urine samples determined the individuals’ 3-month average exposure.

**Results:**

Fair to good reproducibility was observed for the serial measurements of Cd (ICC = 0.53) in spot samples collected during the 3-month period, whereas the serial measurements of As, Co, Cu, Pb, Mo, and Ni showed poor reproducibility (ICCs = 0.01–0.29). Use of single spot urine samples to classify the high (top 33%) 3-month average metal levels had uniformly high specificities (0.70–0.84) but relatively low sensitivities (0.40–0.57), except for Cd (0.77). The minimum number of specimens (k) required to estimate the participant-specific mean for the seven metals within 20% of the “true” values ranged from 3 for Cd to 27 for Ni.

**Conclusions:**

The high variability observed in the urinary levels of As, Co, Cu, Pb, Mo, and Ni suggests that a single measurement provides only a brief snapshot in time of the exposure levels of an individual, which can result in a moderate degree of exposure misclassification.

**Citation:**

Wang YX, Feng W, Zeng Q, Sun Y, Wang P, You L, Yang P, Huang Z, Yu SL, Lu WQ. 2016. Variability of metal levels in spot, first morning, and 24-hour urine samples over a 3-month period in healthy adult Chinese men. Environ Health Perspect 124:468–476; http://dx.doi.org/10.1289/ehp.1409551

## Introduction

Metals, including essential and nonessential elements, are naturally present in the environment at highly varying concentrations and are also widely used in industrial processes and human activities; humans can be exposed to metals either voluntarily through supplementation or involuntarily through intake of contaminated food and water and inhalation of ambient air pollution (Meeker et al. 2010). Consequently, detectable levels of metals have been reported in most human biological samples from numerous studies worldwide including in China [Centers for Disease Control and Prevention (CDC) 2014; Feng et al. 2015; Health Canada 2010; Kile et al. 2009; Smolders et al. 2014]. Toxic elements such as arsenic (As), cadmium (Cd), and lead (Pb) have been associated with impaired human semen quality and sperm DNA integrity at relatively low levels (Meeker et al. 2008; Telisman et al. 2007). Essential elements, such as cobalt (Co), copper (Cu), molybdenum (Mo), and nickel (Ni), are necessary for good health but have also been reported to be associated with changes to male reproductive health measures, such as reduced semen quality and altered serum levels of testosterone if above specific levels in animals and in humans (Danadevi et al. 2003; Lewis and Meeker 2015; Meeker et al. 2008; Pedigo et al. 1988).

Measurements of metals in urine are frequently used as biomarkers in human investigations, especially in surveys involving a large number of participants, such as the National Health and Nutrition Examination Survey (NHANES) in the USA (CDC 2014) and the Canadian Health Measures Survey (CHMS) in Canada (Health Canada 2010). A spot urine sample is the most commonly used sample type because its collection is relatively simple and noninvasive. However, metals in spot samples are likely to vary over time because of the variable exposure driven by changes in diet, lifestyle, or the daily activities of a person (Barr et al. 2005; Smolders et al. 2014). That variation may be more pronounced for metals that are rapidly eliminated after external exposure. Correction for urinary dilution, such as creatinine-corrected concentrations, may introduce an additional source of variability that does not reflect urine dilution (Barr et al. 2005). Because most reproductive health end points of interest, such as sperm quality, are likely associated with metal exposures over time intervals of months, concerns have been raised as to whether single measurements of metals in spot urine samples accurately reflect individual exposure over time (Smolders et al. 2014).

Several previous studies have examined the temporal variability in the urinary levels of As, Cd, and Ni. In a Bangladesh study, Kile et al. (2009) found fair to good reproducibility in urinary As concentrations but poor reproducibility in percent As species among 196 participants who provided repeated spot urine samples during a 2-year period. In a U.S. study conducted with 141 women, Gunier et al. (2013) revealed that Cd concentrations in repeated 24-hr urine samples were only fair to good reproducible over 3–9 months. A more recent study in Belgium collecting spot urine samples from 8 adults also reported poor reproducibility in urinary As and Ni concentrations over a 6-day period (Smolders et al. 2014). Differences in the source and extent of exposure (e.g., smoking status, water consumption, fish intake) may contribute to some of the differences among these studies. However, the variability patterns of metals in spot, first morning, and 24-hr urine samples obtained from the same person during a 3-month period, which corresponds to the duration of spermatogenesis, remain unclear.

To address the data gap, we conducted this study in 11 men with the intention to *a*) assess the reproducibility of serial measurements of As, Cd, Co, Cu, Mo, Ni, and Pb in urine during a 3-month period; *b*) assess the effect of concentration corrections, including creatinine correction, creatinine as a covariate, and the urinary excretion rate (UER) calculation, on the reproducibility of metal measurements; *c*) evaluate the correlation between spot, first morning, and 24-hr urine samples collected within 24 hr; and *d*) evaluate the sensitivity and specificity of spot samples that were used to classify the individuals’ 3-month average excretion measurements.

## Materials and Methods

*Study population and sample collection.* The research protocol was approved by the Ethics Committee of Tongji Medical College. From September 2012 to February 2013, 11 men were recruited to participate in a study designed to examine the variability in urinary levels of dichloroacetic acid and trichloroacetic acid (Wang et al. 2014). The study volunteers were healthy, nonsmoking men living in a restricted geographical area in Wuhan, China, 21 to 28 years of age (mean age, 23.64 ± 1.96 years), with no history of diabetes or renal disease and no documented occupational exposure to metals. Each participant gave written informed consent before participation.

Basic study details have been previously described (Wang et al. 2014). In brief, during the study period of 3 months, 11 participants were asked to provide urine samples on days 0, 1, 2, 3, 4, 30, 60, and 90. The participants collected all urine samples in a trace elements–free polyethylene specimen container on the sampling days. After recording the total volume and time of each void, the urine was decanted in a prelabeled, trace elements–free polyethylene urine cup, and stored at –40°C until analysis (within 6 months). A total of 529 of 535 possible spot urine samples were collected during the study period (6 urine voids were not collected), including 88 first morning urine samples (1 for each study participant on each of the 8 sampling days). The participants had no food or drink restrictions, but they were asked to complete a food diary that recorded detailed information on food consumption during the sampling period.

*Metal determination.* The concentrations of As, Cd, Co, Cu, Mo, Ni, and Pb in the urine were analyzed using a method described in detail in our recent study (Feng et al. 2015). Briefly, a 3.0-mL aliquot of urine was transferred to a polyethylene tube containing 15 μL of 67% (vol/vol) HNO_3_ (Optima^TM^ grade; Fisher), and stored in a refrigerator at 5°C for at least 24 hr. After the urine samples were brought to room temperature (approximately 20°C), 1.0 mL of the sample was diluted to 5.0 mL with 1.2% (vol/vol) HNO_3_ (Optima^TM^ grade), which was then detected using an Agilent 7700x inductively coupled plasma–mass spectrometer with an octopole-based collision/reaction cell (Agilent Technologies). We used the standard reference materials (SRM) 2670a and 1640a (both purchased from National Institute of Standards and Technology, Gaithersburg, MD, USA), as well as a spiked pooled sample of 100 urine samples randomly selected from samples collected for the present study as a quality control. SRM 2670a and spiked urine were analyzed at low and high concentrations for each element after a calibration procedure to evaluate the method’s accuracy. SRM 1640a was measured every 20 samples to ensure instrument performance. The spike recoveries for the seven metals ranged from 85% to 114% and the coefficient of variance (intraday and interday variation) was not higher than 10.00%. Additionally, each batch included one blank urine sampling tube containing 3-mL deionized water, which was analyzed using the same protocols as described for the urine samples. Analyte concentrations were below the limits of quantification (LOQ) in all blank samples. The LOQs for As, Cd, Co, Cu, Mo, Ni, and Pb were 0.013, 0.002, 0.001, 0.234, 0.004, 0.035, and 0.178 μg/L, respectively. Values below the LOQ were assigned to LOQ divided by the square root of 2 for analysis.

Urinary metal concentrations/excretions were expressed in the following three ways: *a*) as an uncorrected concentration (micrograms per liter); *b*) as a creatinine-corrected concentration, calculated by dividing the uncorrected concentration by the urinary creatinine concentration (micrograms per gram creatinine); and *c*) as the UER of each metal, computed by dividing the total mass of excretion by the amount of time since the previous void (micrograms per hour) (Akerstrom et al. 2013). We also adjusted for urine dilution by including creatinine as a model covariate (Kim et al. 2011). Creatinine concentrations were measured using a commercial test kit based on the picric acid assay (Jiancheng Bioengineering Ltd.). We defined spot samples as all individual urine voids collected during a given day, including the first morning samples. We defined first morning urine samples as the first sample collected from an individual at or after 0500 hours each day and 24-hr urine collections as the volume-weighted average of all specimens collected from an individual during a 24-hr period starting at midnight (Wang et al. 2014).

Data analysis. Data analyses were performed using SAS, version 9.2 (SAS Institute Inc., Cary, NC, USA). We first conducted descriptive statistics and assessed normality. Because of the skewed distribution of the urinary metal measurements, log_10_-transformed values were used for subsequent analyses.

We constructed a line chart to visually compare the within-person and between-person variability of urinary metal levels across the 3-month period. To quantitatively assess the variance component of the metals, we further estimated the within-person and between-person variance for each sample type (spot, first morning, and 24-hr urine samples) based on output from multilevel random-effects models. We estimated the between-person, within-person/between-day, and within-person/within-day variances for spot samples; for first morning and 24-hr urine samples, we calculated only the between-person and within-person variances because one value was available each day. We report the variance apportionments of metals separately for samples collected days apart only, for samples collected months apart only, and for all urine samples. The variances for “days apart” samples are based on urine samples collected on days 0, 1, 2, 3, and 4 (5 sampling days total) and variances for “months apart” samples are based on urine samples collected on days 0, 30, 60, and 90 (4 sampling days total). Akaike information criterion (AIC) values (lower AIC values are associated with better models) were calculated to assess the fit of the models (uncorrected concentrations, creatinine-corrected concentrations, creatinine as a covariate and UERs) based on all spot samples (*n* = 529). Intraclass correlation coefficients (ICCs) were computed to assess the reproducibility of repeated measurements. The ICC, ranging from 0 (low reproducibility) to 1 (high reproducibility), is the ratio of the between-person variance to the sum of the between-person and within-person variance. We used standardized criteria proposed by Rosner (2000) to evaluate the ICCs: poor reproducibility (ICC < 0.40), fair to good reproducibility (0.40 ≤ ICC < 0.75), and excellent reproducibility (ICC ≥ 0.75). We also estimated the variance apportionment of creatinine concentrations in the three sample types.

We used Pearson’s correlation analyses to examine the correlation between the metal concentrations measured in a spot (randomly selected each day) or first morning urine sample and the concentrations measured in the same-day 24-hr collection. We also constructed mixed regression models to assess the efficacy of metal concentrations in same-day spot (randomly selected each day) or first morning urine samples as predictors of their 24-hr urine levels. The coefficient of determination (*R* ^2^) was estimated to assess the predictive power of the model.

We finally evaluated how well 1, 2, or 3 randomly selected spot urine samples collected on different days could correctly classify the participants into the high (top 33%) exposure group based on their 3-month average measurements by comparing the distribution of “true” and “predicted” levels for agreement according to the method described by Bradman et al. (2013). For the “true” exposure, we calculated the arithmetic mean of metal concentrations (log_10_ scale) for each person using all his spot samples (including first morning samples) collected during the 3-month period, categorized the values into tertiles, and identified the “true” top 33% exposure levels. For the “predicted” levels, we created 10 data sets each containing 1 randomly selected urine sample per person. Within each randomly-selected data set, we categorized the 11 randomly selected concentrations into tertiles, and also identified the “predicted” top 33% exposure levels. We reported the average sensitivity and specificity observed across the 10 separate random samples. To determine whether collection of repeated urine samples improved the accuracy of classification, we repeated the analysis using the arithmetic mean of the log_10_-transformed metal concentrations in 2 or 3 urine samples randomly selected from each participant collected on different days. The sensitivity and specificity were separately calculated for all spot samples and for first morning urine samples only. We further calculated the minimum number of spot samples (including first morning samples) (*k*) needed to estimate the participant-specific mean for metals within 20% of the ‘‘true’’ values with a probability of 95% after a log_10_ transformation without regard to the day of sample collection, using the equation: *k* = (1.96 × CV/20)^2^, where CV is the within-person coefficient of variation (Kim et al. 2014).

## Results

The basic characteristics of the 11 participants are presented in Supplemental Material, Table S1. Three participants reported eating fish on one occasion during the 3-month sampling period; the other participants did not eat any fish. None of the participants ate seafood other than fish during the sampling period. The participants urinated between 3 and 11 times per day (mean, 6.01 voids), and the volume of voids ranged from 20.3 to 740.6 mL (mean, 195.4 mL). The distribution of metal concentrations in spot, first morning, and 24-hr urine samples is presented in Supplemental Material, Table S2. All seven metals were detected in > 95% of the 529 spot samples.

**Figure 1:**
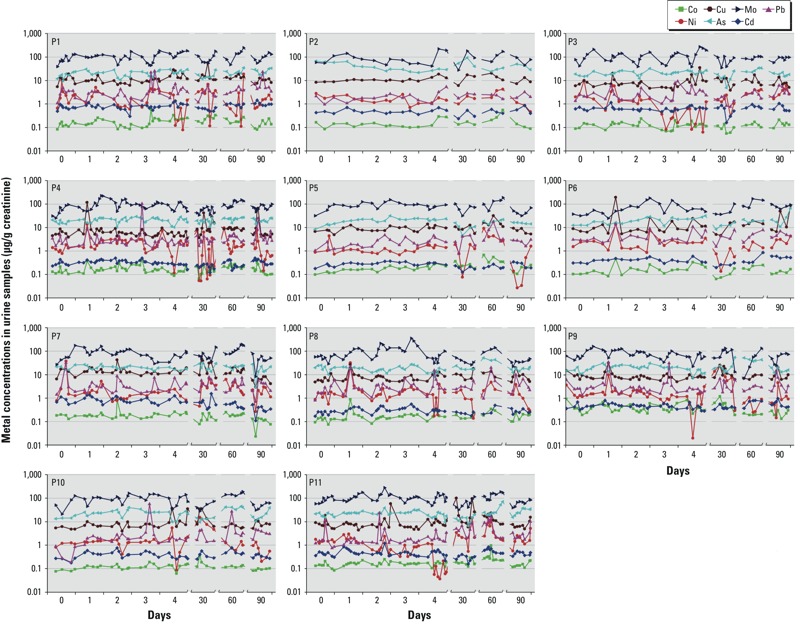
Creatinine-corrected metal concentrations (μg/g creatinine) in the urine collected from 11 men on 8 days during a 3-month period. Each graph represents an individual participant (labeled P1–P11). The dots in each graph represent the metal concentrations in each spot urine sample (including first morning samples) collected on each sampling day.

Figure 1 presents the creatinine-corrected concentrations of metals (micrograms per gram creatinine) in spot urine samples collected from the 11 men on 8 days during the 3-month period. There were no pronounced cyclic patterns in the metal concentrations among the 11 participants, although the line charts showed that some metal concentrations in urine varied up to two (e.g., As, Co, Mo) or three (e.g., Cu, Ni, Pb) orders of magnitude during the period. The concentrations of Cd in spot samples were rather consistent and varied within an order of magnitude for most participants (P2, P3, P4, P5, P6, P8, P9, P10, P11; Figure 1).

We used four different models to evaluate the influence of concentration corrections on the reproducibility of metal measurements in spot samples (Table 1). The highest AIC values for the multilevel random-effects models based on all spot urine samples were achieved with the uncorrected values for most metals, which indicated the worst fit of the models. The AIC decreased if we accounted for urinary dilution. The apportionments of the within-person and between-person variances for metal measurements were similar based on the creatinine-corrected, creatinine as a covariate, and UER models. Specifically, we found that accounting for urinary dilution increased the between-person relative to the within-person variance for Cd and Co but decreased the variance for As and Cu. The best model fit was achieved with the creatinine-corrected model for Co, Mo, and Ni, and the creatinine as a covariate model for As, Cd, Cu, and Pb. Because the variance apportionments of metals estimated from both models were similar and the 24-hr voids data cannot account for urine dilution by modeling creatinine as a covariate, we used only the creatinine-corrected values in subsequent analyses.

**Table 1:** The variance apportionment of log_10_-transformed metal concentrations/excretions in spot samples collected from 11 adults (*n *= 529).

Type of correction for urinary dilution	As	Cd	Co	Cu	Pb	Mo	Ni
Uncorrected (μg/L)
AIC^*a*^	700.7	496.9	404.4	402.8	401.3	730.1	1106.0
Between persons σ^2^ (%)^*b*^	0.052 (67)	0.048 (25)	0.043 (25)	0.033 (21)	0.008 (6)	0.011 (4)	0.001 (1)
Within person, between day σ^2^ (%)^*c*^	0.025 (32)	0.020 (10)	0.025 (15)	0.015 (13)	0.031 (22)	0.039 (16)	0.175 (31)
Within person, within day σ^2^ (%)^*d*^	0.001 (1)	0.126 (65)	0.102 (60)	0.107 (69)	0.102 (72)	0.198 (80)	0.376 (68)
Creatinine-corrected (μg/g creatinine)
AIC^*a*^	204.6	278.1	16.6	314.0	570.1	239.7	965.8
Between persons σ^2^ (%)^*b*^	0.012 (19)	0.046 (53)	0.027 (29)	0.010 (8)	0.009 (5)	0.002 (2)	0.001 (1)
Within person, between day σ^2^ (%)^*c*^	0.026 (39)	0.016 (18)	0.022 (24)	0.020 (17)	0.023 (13)	0.038 (34)	0.163 (36)
Within person, within day σ^2^ (%)^*d*^	0.028 (42)	0.025 (29)	0.043 (47)	0.088 (75)	0.149 (82)	0.071 (64)	0.281 (63)
Creatinine as a covariate
AIC^*a*^	31.3	64.8	34.5	204.0	359.3	369.2	980.4
Between persons σ^2^ (%)^*b*^	0.009 (11)	0.040 (42)	0.030 (32)	0.013 (13)	0.005 (4)	0.001 (1)	0.001 (1)
Within person, between day σ^2^ (%)^*c*^	0.024 (30)	0.017 (18)	0.021 (23)	0.017 (17)	0.027 (21)	0.037 (28)	0.156 (35)
Within person, within day σ^2^ (%)^*d*^	0.047 (59)	0.039 (40)	0.041 (55)	0.070 (70)	0.094 (75)	0.094 (71)	0.289 (64)
Urinary excretion rate (μg/hr)
AIC^*a*^	248.6	223.9	351.7	547.6	705.4	495.7	1027.5
Between persons σ^2^ (%)^*b*^	0.019 (16)	0.051 (38)	0.045 (29)	0.012 (7)	0.006 (3)	0.008 (5)	0.001 (1)
Within person, between day σ^2^ (%)^*c*^	0.023 (20)	0.011 (8)	0.013 (8)	0.010 (6)	0.017 (8)	0.031 (19)	0.159 (33)
Within person, within day σ^2^ (%)^*d*^	0.075 (64)	0.074 (54)	0.096 (63)	0.149 (87)	0.201 (89)	0.124 (76)	0.322 (66)
σ^2^ = variance. ^***a***^Akaike information criterion (AIC) values were used to compare the fit of models. ^***b***^The proportion of between-person variance to the total variance. ^***c***^The proportion of within-person between-day variance to the total variance. ^***d***^The proportion of within-person within-day variance to the total variance.							

The within-person and between-person variance apportionments of creatinine-corrected metal concentrations in spot, first morning, and 24-hr urine samples collected from 11 adults on 8 days during the 3-month period are shown in Table 2. Fair to good reproducibility was observed for serial measurements of Cd (ICC = 0.53) in spot samples during the 3-month period, whereas serial measurements of As, Co, Cu, Pb, Mo, and Ni showed poor reproducibility (ICCs = 0.01–0.29). For As, Co, Cu, Pb, Mo, and Ni, the total within-person variance (between-day + within-day) (71–99%) was much higher than the between-person variance (1–29%), and the within-day (42–82%) variance was the largest component of the total variance. Compared with spot urine samples, the serial measurements of Cd (ICC = 0.68 and 0.70 for first morning and 24-hr urine samples, respectively) and Co (ICC = 0.55 and 0.50 for first morning and 24-hr urine samples, respectively) in first morning and 24-hr urine samples provided apparently higher consistency; however, the advantages of these two sample types over spot samples were not as evident for the other metals. We reanalyzed the data by including “time since the previous urination” as a model covariate and found that the estimated variance apportionments for the metals in all spot samples remained largely unchanged (percentage differences in estimated variance apportionments between models ranged from 0.0% to 37.5%).

**Table 2:** The variance apportionment of log_10_-transformed creatinine-corrected metal concentrations in the three sample types collected from 11 adults during a 3-month period.

Type of sample	As	Cd	Co	Cu	Pb	Mo	Ni
Spot sample (*n *= 529)
ICC	0.19	0.53	0.29	0.08	0.05	0.02	0.01
Between persons σ^2^ (%)^*a*^	0.012 (19)	0.046 (53)	0.027 (29)	0.010 (8)	0.009 (5)	0.002 (2)	0.001 (1)
(95% CI)^*b*^	(0.000, 0.025)	(0.010, 0.082)	(0.004, 0.050)	(0.000, 0.021)	(–0.003, 0.021)	(–0.004, 0.008)	(–0.005, 0.007)
Within person, between day σ^2^ (%)^*c*^	0.026 (39)	0.016 (18)	0.022 (24)	0.020 (17)	0.023 (13)	0.038 (34)	0.163 (36)
(95% CI)^*b*^	(0.018, 0.034)	(0.011, 0.021)	(0.014, 0.030)	(0.011, 0.031)	(0.010, 0.036)	(0.025, 0.051)	(0.111, 0.217)
Within person, within day σ^2^ (%)^*d*^	0.028 (42)	0.025 (29)	0.043 (47)	0.088 (75)	0.149 (82)	0.071 (64)	0.281 (63)
(95% CI)^*b*^	(0.025, 0.031)	(0.022, 0.028)	(0.038, 0.048)	(0.078, 0.098)	(0.132, 0.165)	(0.063, 0.079)	(0.248, 0.310)
First morning sample (*n *= 88)^*e*^
ICC	0.28	0.68	0.55	0.20	0.14	0.02	0.01
Between person σ^2^ (%)^*a*^	0.016 (28)	0.059 (68)	0.059 (55)	0.015 (20)	0.011 (14)	0.002 (2)	0.001 (1)
(95% CI)^*b*^	(0.001, 0.031)	(0.014, 0.102)	(0.011, 0.107)	(–0.001, 0.032)	(–0.004, 0.026)	(–0.009, 0.013)	(–0.007, 0.011)
Within person σ^2^ (%)^*c*^	0.041 (72)	0.027 (32)	0.049 (45)	0.059 (80)	0.069 (86)	0.094 (98)	0.220 (99)
(95% CI)^*b*^	(0.030, 0.052)	(0.020, 0.034)	(0.036, 0.062)	(0.043, 0.075)	(0.051, 0.087)	(0.069, 0.119)	(0.166, 0.274)
24-hr collection (*n *= 88)^*e*^
ICC	0.29	0.70	0.50	0.12	0.07	0.02	0.01
Between person σ^2^ (%)^*a*^	0.013 (29)	0.048 (70)	0.034 (50)	0.009 (12)	0.006 (7)	0.001 (2)	0.001 (1)
(95% CI)^*b*^	(–0.112, 0.138)	(0.012, 0.087)	(0.006, 0.062)	(–0.004, 0.022)	(–0.006, 0.018)	(–0.056, 0.059)	(–0.067, 0.070)
Within person σ^2^ (%)^*c*^	0.032 (71)	0.021 (30)	0.034 (50)	0.065 (88)	0.079 (93)	0.049 (98)	0.153 (91)
(95% CI)^*b*^	(0.024, 0.040)	(0.016, 0.026)	(0.024, 0.044)	(0.047, 0.083)	(0.058, 0.100)	(0.036, 0.062)	(0.115, 0.191)
σ^2^ = variance. ^***a***^The proportion of between-person variance to the total variance. ^***b***^Confidence intervals for the variance components. ^***c***^The proportion of within-person between-day variance to the total variance. ^***d***^The proportion of within-person within-day variance to the total variance. ^***e***^For first morning and 24-hr urine collections, the distinction between within-day vs. between-day variability is not applicable with only one measurement per day.							

We also separately calculated the variance apportionments of creatinine-corrected metal concentrations in the three sample types based on samples collected days apart (including samples collected on days 0, 1, 2, 3, and 4; total *n* = 326) and months apart (including samples collected on days 0, 30, 60, and 90; total *n* = 265) (Table 3). If urine samples were collected days apart, fair to good reproducibility was obtained for As (ICC = 0.41), Cd (ICC = 0.64), and Co (ICC = 0.41) in spot samples; however, poor reproducibility (ICCs = 0.01–0.18) was observed for the other metals. Compared with spot urine samples, higher ICCs were observed for Cd (0.77), Co (0.74), Cu (0.44), and Pb (0.36) in first morning urine samples, as well as Cd (0.82) and Co (0.69) in 24-hr collections. For urine samples collected months apart, decreased ICCs were observed for the seven metals in each sample type, and fair to good reproducibility was obtained only for Cd (ICCs = 0.51–0.65). The reproducibility of creatinine in spot samples within individuals was poor over periods ranging from days to months (ICCs = 0.09–0.15), but was fair to good in first morning and 24-hr urine samples (ICCs = 0.49–0.62) (see Supplemental Material, Table S3).

**Table 3:** The variance apportionment of log_10_-transformed creatinine-corrected metal concentrations in urine samples, collected days apart and months apart, from 11 adults.

Type of sample	As	Cd	Co	Cu	Pb	Mo	Ni
Days apart (*n* = 326)
Spot sample
ICC	0.41	0.64	0.41	0.18	0.10	0.01	0.12
Between persons σ^2^ (%)^*a*^	0.011 (41)	0.047 (64)	0.033 (41)	0.017 (18)	0.018 (10)	0.001 (1)	0.012 (3)
Within person, between day σ^2^ (%)^*b*^	0.015 (56)	0.007 (10)	0.007 (9)	0.012 (12)	0.011 (6)	0.024 (25)	0.107 (31)
Within person, within day σ^2^ (%)^*c*^	0.001 (3)	0.020 (26)	0.041 (50)	0.068 (70)	0.161 (84)	0.070 (74)	0.232 (66)
First morning sample^*d*^
ICC	0.32	0.77	0.74	0.44	0.36	0.01	0.12
Between person σ^2^ (%)^*a*^	0.016 (32)	0.064 (77)	0.077 (74)	0.017 (44)	0.022 (36)	0.001 (1)	0.013 (12)
Within person σ^2^ (%)^*b*^	0.034 (68)	0.019 (23)	0.027 (26)	0.022 (56)	0.040 (64)	0.080 (99)	0.094 (88)
24-hr collection^*d*^
ICC	0.34	0.82	0.69	0.16	0.10	0.06	0.14
Between person σ^2^ (%)^*a*^	0.011 (34)	0.049 (82)	0.040 (69)	0.011 (16)	0.010 (10)	0.002 (6)	0.016 (14)
Within person σ^2^ (%)^*b*^	0.021 (66)	0.011 (18)	0.018 (31)	0.057 (84)	0.089 (90)	0.043 (96)	0.100 (86)
Months apart (*n* = 265)
Spot sample
ICC	0.21	0.51	0.18	0.01	0.01	0.01	0.01
Between persons σ^2^ (%)^*a*^	0.021 (21)	0.051 (51)	0.019 (18)	0.001 (1)	0.001 (1)	0.001 (1)	0.001 (1)
Within person, between day σ^2^ (%)^*b*^	0.041 (42)	0.021 (21)	0.044 (43)	0.031 (23)	0.048 (25)	0.045 (36)	0.185 (36)
Within person, within day σ^2^ (%)^*c*^	0.036 (37)	0.029 (28)	0.041 (39)	0.102 (76)	0.137 (74)	0.078 (63)	0.318 (63)
First morning sample^*d*^
ICC	0.29	0.59	0.31	0.10	0.11	0.09	0.01
Between person σ^2^ (%)^*a*^	0.024 (29)	0.051 (59)	0.035 (31)	0.011 (10)	0.011 (11)	0.001 (9)	0.001 (1)
Within person σ^2^ (%)^*b*^	0.058 (71)	0.036 (31)	0.078 (69)	0.096 (90)	0.090 (89)	0.113 (99)	0.322 (99)
24-hr collection^*d*^
ICC	0.30	0.65	0.30	0.01	0.01	0.02	0.01
Between person σ^2^ (%)^*a*^	0.021 (30)	0.051 (65)	0.025 (30)	0.001 (1)	0.001 (1)	0.001 (2)	0.001 (1)
Within person σ^2^ (%)^*b*^	0.050 (70)	0.027 (35)	0.058 (70)	0.073 (99)	0.086 (99)	0.054 (98)	0.176 (99)
Days apart: days 0, 1, 2, 3, and 4; months apart: days 0, 30, 60, and 90. σ^2^ = variance. ^***a***^The proportion of between-person variance to the total variance. ^***b***^The proportion of within-person between-day variance to the total variance. ^***c***^The proportion of within-person within-day variance to the total variance. ^***d***^For first morning and 24-hr urine collections, the distinction between within-day vs. between-day variability is not applicable with only one measurement per day.							

**Figure 2:**
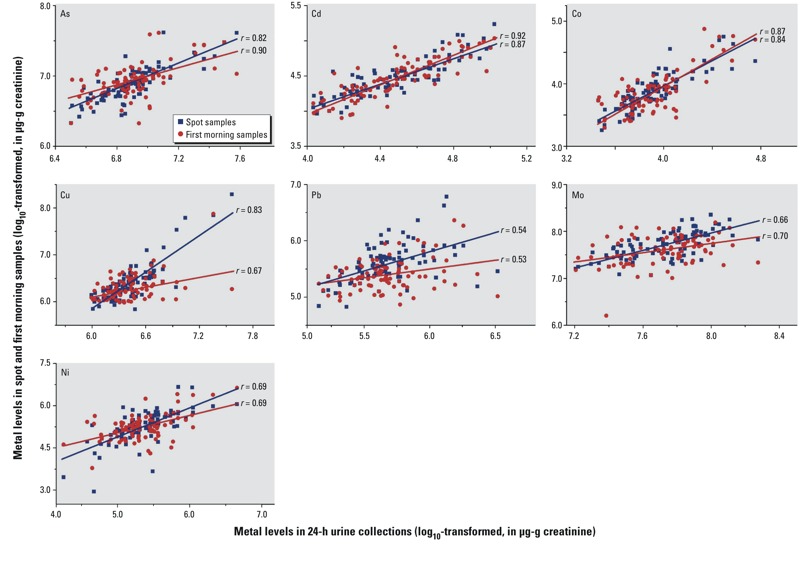
Scatter plots of correlations (*r*) between creatinine-corrected metal concentrations (log_10_-transformed) in a 24-hr collection and the concentrations measured in a spot sample (randomly selected) or a first morning sample within the same 24-hr period (*n* = 88). Lines represent the linear trend (all *p*-values < 0.001).

Scatter plots of correlations between creatinine-corrected metal concentrations in the three sample types collected within the same-day 24-hr period are presented in Figure 2. The creatinine-corrected metal concentrations in a spot or first morning urine sample were significantly correlated with those measured in the same-day 24-hr urine collection (all *p*-values < 0.001). The abilities of same-day spot or first morning urine samples to predict 24-hr urine metal excretions based on mixed regression models are presented in Table 4. For models examining a spot sample and its respective 24-hr sample, the predictive power of the models was high for Cd (*R* ^2^ = 0.76) and Co (*R* ^2^ = 0.71) but only low-to-moderate for the other metals (*R* ^2^ = 0.29–0.69). Apparent advantage of using a first morning urine sample rather than a spot sample to predict the respective 24-hr collection was observed for only As (*R* ^2^ = 0.81 for first morning samples vs. 0.67 for spot samples).

**Table 4:** Creatinine-corrected models of 24-hr metal excretions using same-day spot or first morning urine samples as predictors.*^a^*

Type of sample	Mixed regression model			Pearson correlation	
	β (95% CI)	Intercept	Estimated *R*^*2c*^	*r*^*d*^	*p*-Value^*d*^
Spot sample (*n* = 88)^*b*^
As	0.66 (0.55, 0.76)*	2.38	0.67	0.82	< 0.001
Cd	0.63 (0.52, 0.75)*	1.66	0.76	0.87	< 0.001
Co	0.63 (0.52, 0.74)*	1.45	0.71	0.84	< 0.001
Cu	0.55 (0.47, 0.63)*	2.92	0.69	0.83	< 0.001
Pb	0.46 (0.31, 0.61)*	3.10	0.29	0.54	< 0.001
Mo	0.51 (0.39, 0.64)*	3.78	0.44	0.66	< 0.001
Ni	0.47 (0.36, 0.57)*	2.90	0.48	0.69	< 0.001
First morning sample (*n* = 88)
As	0.79 (0.70, 0.88)*	1.44	0.81	0.90	< 0.001
Cd	0.79 (0.70, 0.88)*	0.96	0.85	0.92	< 0.001
Co	0.69 (0.60, 0.78)*	1.26	0.76	0.87	< 0.001
Cu	0.70 (0.54, 0.86)*	2.02	0.45	0.67	< 0.001
Pb	0.55 (0.36, 0.74)*	2.71	0.28	0.53	< 0.001
Mo	0.50 (0.39, 0.62)*	3.91	0.49	0.70	< 0.001
Ni	0.57 (0.44, 0.70)*	2.35	0.48	0.69	< 0.001
^***a***^Spot and first morning urine samples were analyzed on the log_10_ scale. ^***b***^Spot urine samples were randomly collected from 11 persons on each day. ^***c***^Estimated from single variable linear regression model. ^***d***^Estimated from Pearson’s correlation analyses. *p < 0.001.					

The results from sensitivity and specificity analysis of the ability to correctly classify the participants into a high-exposure group (3-month average concentrations) based on 1, 2, or 3 randomly selected urine samples collected on different days are presented in Table 5. The proportion of participants who had the highest 3-month average exposure (top 33%) that would be correctly classified as such using a single urine sample (the sensitivities) was high for Cd (0.77 for spot samples and 0.83 for first morning samples) but relatively low for the other metals (range, 0.37–0.57). Use of two spot samples collected days apart provided ≤ 10% increase in sensitivities for most metals. If three spot urine samples were collected days apart, moderate-to-high sensitivities in the range of 0.63–0.97 were obtained for As, Cd, Co, Cu, Pb, and Mo. Within the first morning sample group, using two samples collected days apart to classify the individual’s exposure provided ≥ 15% increase in sensitivities over a single sample for As, Co, Cu, and Ni; if three samples were collected days apart, moderate-to-high sensitivities in the range of 0.60–0.97 were observed for As, Cd, Co, Cu, Mo, and Ni. The specificity was fair to good and uniformly higher than the sensitivity in each type of sample collection. The collection of urine samples months apart did not offer an advantage in exposure classification over samples collected days apart. The minimum number of specimens (*k*) required to estimate the participant-specific mean for the seven metals within 20% of the ‘‘true’’ values ranged from 3 for Cd to 27 for Ni (Table 5).

**Table 5:** Sensitivity and specificity*^a^* for classifying participants with high (top 33%) 3-month average metal concentrations*^b^* with 1, 2, and 3 urine samples.

Type of sample collection	Sensitivity (specificity)						
	As	Cd	Co	Cu	Pb	Mo	Ni
Spot sample
1 sample	0.57 (0.75)	0.77 (0.84)	0.57 (0.80)	0.53 (0.78)	0.50 (0.73)	0.47 (0.71)	0.40 (0.70)
2 samples^*c*^ (days apart)	0.60 (0.80)	0.97 (0.93)	0.60 (0.74)	0.63 (0.80)	0.57 (0.78)	0.50 (0.69)	0.53 (0.75)
2 samples^*c*^ (months apart)	0.63 (0.75)	0.80 (0.81)	0.67 (0.76)	0.67 (0.78)	0.57 (0.75)	0.60 (0.78)	0.43 (0.74)
3 samples^*c*^ (days apart)	0.63 (0.76)	0.97 (0.91)	0.67 (0.78)	0.73 (0.79)	0.70 (0.76)	0.67 (0.79)	0.50 (0.70)
3 samples^*c*^ (months apart)	0.63 (0.73)	0.87 (0.88)	0.80 (0.81)	0.70 (0.78)	0.63 (0.78)	0.63 (0.75)	0.67 (0.78)
*k*^*d*^	4	3	5	13	20	6	27
First morning sample
1 sample	0.40 (0.69)	0.83 (0.88)	0.57 (0.84)	0.50 (0.73)	0.37 (0.78)	0.47 (0.70)	0.43 (0.74)
2 samples^*c*^ (days apart)	0.63 (0.75)	0.97 (0.95)	0.73 (0.81)	0.67 (0.76)	0.33 (0.71)	0.40 (0.66)	0.67 (0.79)
2 samples^*c*^ (months apart)	0.50 (0.73)	0.97 (0.98)	0.70 (0.81)	0.40 (0.70)	0.43 (0.71)	0.67 (0.78)	0.27 (0.65)
3 samples^*c*^ (days apart)	0.70 (0.78)	0.97 (0.98)	0.70 (0.78)	0.73 (0.80)	0.47 (0.74)	0.60 (0.73)	0.73 (0.80)
3 samples^*c*^ (months apart)	0.53 (0.73)	0.97 (0.98)	0.83 (0.83)	0.30 (0.64)	0.43 (0.68)	0.73 (0.76)	0.20 (0.58)
Days apart: days 0, 1, 2, 3, and 4; months apart: days 0, 30, 60, and 90. ^***a***^Average sensitivity and specificity was computed based on 10 data sets each containing one randomly selected urine sample per person. ^***b***^Calculations use creatinine-corrected metal concentrations (μg/g creatinine) on the log_10_ scale. ^***c***^Urine samples were collected on different days. ^***d***^The minimum number of spot samples (including first morning samples) required to estimate the participant-specific mean within 20% of the “true” value without regard to the day of sample collection.							

## Discussion

In the present study we found substantial within-person variation in the serial measurements of metals in urine samples in 11 men during a 3-month period. We obtained fair to good reproducibility for the creatinine-corrected As, Cd, and Co concentrations in spot samples collected on 5 consecutive days (ICCs = 0.41–0.64), suggesting that urinary As, Cd, and Co concentrations were fairly stable over the short term. For Cu, Pb, Mo, and Ni, however, relatively low ICCs in the range of 0.01–0.18 were observed. The ICCs of the metals decreased if urine samples were collected further apart in time, and fair to good reproducibility was observed for only Cd (ICC = 0.51). The decline in ICCs is not unexpected because the collection of samples months apart includes variability in urinary metal concentrations that contributed to both day-to-day changes and monthly trends in metal exposure, such as seasonal variation in diet, lifestyle, and activity patterns, as well as other environmental or biologic factors (Hauser et al. 2004). However, in this study, the collection of urine samples months apart did not offer an advantage in exposure classification over samples collected days apart, which suggests that urine samples collected days apart may be as good as samples collected months apart.

Twenty-four–hour urine samples have been recommended by some as the “gold standard” for evaluating personal exposure to toxic chemicals that are excreted mainly through the urine [Bradman et al. 2013; U.S. Environmental Protection Agency (EPA) 1996]. In this study, although metal concentrations in spot urine samples were significantly correlated with those in the same-day 24-hr urine samples, only low-to-moderate predictive power was achieved for As, Cu, Pb, Mo, and Ni (*R* ^2^ = 0.29–0.69) using creatinine-corrected spot urine samples as predictors. This finding implies that measurements of these metals in a single spot urine sample may not be good surrogates for those in a 24-hr collection. Many studies have instead used first morning urine samples to evaluate personal exposure because they are more concentrated and correlated with the 24-hr urine collection (Kissel et al. 2005; Scher et al. 2007). However, in the present study, after correction for urine dilution, the levels of metal in spot and first morning samples were similar, and the advantage of using a first morning urine sample rather than a spot sample to predict the respective 24-hr collection was evident only for As. Moreover, compared with spot samples, the use of first morning and 24-hr urine collections improved the consistency of serial measurements only for Cd and Co. The high between-day (within-person) variance of As, Cu, Pb, Mo, and Ni in first morning and 24-hr urine samples (71–99%) suggests that concentrations in a single first morning or 24-hr urine sample may not provide accurate estimates of individual exposures over weeks or months.

Our findings support the use of creatinine-corrected metal concentrations to control for urinary dilution among adult men. Our findings suggest that the differences in urine dilution most likely explained some of the observed variation in concentrations of most metals because the model fits improved if we accounted for urine dilution. Consistent with this notion, we found rather poor reproducibility of urinary creatinine concentrations in spot samples within individuals. The UER calculation, which is a measure of the chemical mass in the sample, has been proposed as an appropriate method to eliminate the variability in urine concentrations from urine dilution (Barr et al. 2005). The similar apportionment of the within-person and between-person variance for metals based on creatinine-corrected and UER models suggests that other sources of variability introduced by creatinine correction (e.g., age, body size, muscle mass) may not be major contributing factors to the observed variation, which reflects true variations in metal excretions. Alternatively, when we included creatinine as a model covariate instead of creatinine-corrected values to account for urine dilution, the variance apportionments of metals remained largely unchanged. The apportionments of variance were also robust to the adjustment for “time since the previous urination” (data not shown), which was proposed as an important parameter to take into account when considering the variability of urinary metal measures (Smolders et al. 2014). However, we did not quantify specific gravity in these samples. Additional research is needed to confirm whether our findings are valid for adult men using specific gravity to account for urinary dilution.

The reproducibility of serial measurements of metal in urine may be affected by the kinetics of absorption, distribution, metabolism, and elimination of the analyte, particularly the relationship between elimination half-life and the intervals between exposure events (Aylward et al. 2014). The possible reason that urinary Cd concentrations were fairly stable during a 3-month period could be related to frequent exposure and Cd’s long biological elimination half-life. Cd is stored in the liver and kidneys with a half-life of 10–30 years and therefore can easily reach a steady state from chronic exposure via ingestion (Gunier et al. 2013). Fair-to-excellent reproducibility of urinary levels of Cd has been revealed in two previous studies of 296 women who collected repeated 24-hr urine samples over 3–9 months (creatinine-corrected ICC = 0.42) (Gunier et al. 2013) and of 8 adults who provided spot urine samples during a 6-day period (creatinine-corrected ICC = 0.75) (Smolders et al. 2014). Serial measurements of other metals were more variable than those of Cd throughout the day and across days, most likely due to their much shorter elimination half-life (Aylward et al. 2014). In support of our findings, Smolders et al. (2014) also reported poor reproducibility for the urine levels of As (creatinine-corrected ICC = 0.21). However, our findings differed from those of Navas-Acien et al. (2009), who found urinary As concentrations were fairly stable during 10 years in 60 U.S. adults with stable environmental exposure to inorganic arsenic in drinking water; and those of Smolders et al. (2014), who observed fair to good reproducibility for serial measurements of Ni (creatinine-corrected ICC = 0.68) among four couples who consumed fish frequently. The varied ICCs between studies may be attributable in part to the differences in duration between repeat urine sample collections (Smolders et al. 2014). Study design, source, and extend of exposure, sex, age, the underlying physiology of the participants and geography where interaction with sources of exposure may differ can also partly explain the variation (Smolders et al. 2014).

In our sensitivity and specificity analysis, we observed trends similar to those of the ICCs. For example, use of single spot urine samples to classify the high (top 33%) 3-month average metal levels had high sensitivity for Cd (0.77) but relatively low sensitivities for the other metals (0.40–0.57). This finding implies that in relying on a single urine sample to categorize an individual’s average exposure levels of As, Co, Cu, Mo, Ni, and Pb over periods of days or months, there is likely exposure misclassification that tends to attenuate risk estimations. In our study population, 3 specimens collected at different times were necessary to estimate participant-specific means within 20% of the ‘‘true’’ values for Cd; whereas more than 27 specimens were required for Ni, which is likely a consequence of poorer reliability secondary to lower concentrations (Kim et al. 2014). However, the calculated sensitivities and specificities may be slightly overestimated because we included “predicted” values in the calculation of the “true” values. A portion of the increased sensitivities and specificities observed when selecting two or three samples for each subject instead of a single sample may also be caused by the increased dependence between the errors of the “predicted” and “true” values (Hauser et al. 2004).

The strengths of the present study include its large sample size per subject and serial measurements of metals in three types of urine samples over durations relevant to male reproductive health. The median urinary levels of metals in this study are similar to those reported in previous studies among Chinese adults (Feng et al. 2015). However, our median urinary levels of As (19.86 μg/L) and Pb (2.75 μg/L) were much higher than those reported for the males from NHANES in the United States (CDC 2014) (8.80 μg/L for As; 0.54 μg/L for Pb) and CHMS in Canada (Health Canada 2010) (12.62 μg/L for As; 0.60 μg/L for Pb), indicating higher As and Pb exposure in our population that may reduce the contribution of the within-person variance to the total variance. In addition, it is likely that between-person variability in our study population of healthy nonsmoking young adult men, who did not use dietary supplements and only rarely consumed fish, was lower than in the general population. Moreover, our findings may not be generalizable to other study populations, such as children or pregnant women, because differences in exposure patterns (e.g., related to diet and lifestyle), and possibly age- and sex-related differences in metabolism, will contribute to variation (Aylward et al. 2014; Smolders et al. 2014). Finally, we did not analyze duplicate aliquots to assess variability due to analytic factors, which may bias ICCs as an estimate of between-person variability within our study population.

## Conclusions

In our study population of adult nonsmoking Chinese men, serial measures of Cd in spot urine samples had fair to good reproducibility over a 3-month period. However, the serial measures of As, Co, Cu, Pb, Mo, and Ni showed poor reproducibility, and the use of single spot samples to classify the individuals’ 3-month average exposure can result in a moderate degree of exposure misclassification. Between-day variance of As, Cu, Pb, Mo, and Ni based on a single first morning void or a single 24-hr urine sample was similar to between-day variance based on a single spot sample, which suggests that improvements in long-term exposure estimation may not offset the additional effort required to collect first morning or 24-hr samples. Collection of multiple urine samples from each study participant is an option for reducing exposure classification errors, and it is valuable to consider the number of specimens required to estimate participant-specific mean values to maximize study efficiency. However, our study does not allow concluding that urine samples collected further apart in time offer an advantage in exposure classification.

## Supplemental Material

(255 KB) PDF
