# Supplementary material for: Variability of Metal Levels in Spot, First Morning, and 24-Hour Urine Samples over a 3-Month Period in Healthy Adult Chinese Men
Source: Environ Health Perspect. 2015 Sep 15;124(4):468–76. doi: 10.1289/ehp.1409551 (PMC4829977; doi:10.1289/ehp.1409551)
Supplement: (255 KB) PDF [file ehp.1409551.s001.acco.pdf]

Environ Health Perspect
DOI: 10.1289/ehp.1409551

Note to Readers: *EHP* strives to ensure that all journal content is accessible to all readers. However, some figures and Supplemental Material published in *EHP* articles may not conform to 508 standards due to the complexity of the information being presented. If you need assistance accessing journal content, please contact ehp508@niehs.nih.gov. Our staff will work with you to assess and meet your accessibility needs within 3 working days.

Supplemental Material

Variability of Metal Levels in Spot, First Morning, and 24-Hour Urine Samples over a 3-Month Period in Healthy Adult Chinese Men

Yi-Xin Wang, Wei Feng, Qiang Zeng, Yang Sun, Peng Wang, Ling You, Pan Yang, Zhen Huang, Song-Lin Yu, and Wen-Qing Lu

Table of Contents

Table S1. Basic characteristics of the 11 men during the sampling days.

Table S2. Uncorrected (μg/L), creatinine-corrected (μg/g creatinine) and urinary excretion rate (UER) (μg/h) of metals in spot, first morning and 24-h urine samples collected from 11 men.

Table S3. The variance apportionment of $\log_{10}$-transformed creatinine concentrations in the three sample types collected from 11 adults.

Table S1. Basic characteristics of the 11 men during the sampling days.

**Characteristics**	P1	P2	P3	P4	P5	P6	P7	P8	P9	P10	P11
Age (years)	28	25	24	23	24	23	24	24	21	21	23
Height (m)	169	178	170	168	177	173	173	175	173	178	177
Wight (kg)	54	72	55	67	58	70	65	68	75	70	66
BMI (kg/m^2)	18.9	22.7	19.0	23.7	18.5	23.4	21.7	22.2	25.1	22.1	21.1
Profession	PhD candidate	PhD candidate	Graduate	Graduate	Graduate	Graduate	Graduate	Graduate	Undergraduate	Undergraduate	Graduate
Smoking	No	No	No	No	No	No	No	No	No	No	No
Dietary supplements	No	No	No	No	No	No	No	No	No	No	No
Seafood consumption[a]	No	No	No	No	No	No	No	No	No	No	No
Fish consumption	No	Once	No	No	No	No	No	Once	No	Once	No
Average volume of voids (mL)	164.1	212.3	230.9	117.0	249.2	295.6	159.9	178.7	198.3	220.9	219.8
Average frequency of voids (times/day)[b]	6.9	3.0	5.8	9.1	4.5	4.1	6.4	6.1	7.0	5.3	8.0

Graduate: Graduate student. [a]Seafood does not include fish. [b]A total of 529 spot urine samples were finally collected (6 missing spot samples), including 88 first morning urine samples.

Table S2. Uncorrected (µg/L), creatinine-corrected (µg/g creatinine) and urinary excretion rate (UER) (µg/h) of metals in spot, first morning and 24-h urine samples collected from 11 men.

**Exposure variables**	**Spot sample (*n*=529)**			**First morning sample (*n*=88)**			**24-h collection (*n*=88)**		
	Mean	Median	Interquartile	Mean	Median	Interquartile	Mean	Median	Interquartile
**Uncorrected**									
As	25.71	19.86	11.11-34.63	41.95	36.69	22.00-48.90	26.13	20.38	17.25-25.32
Cd	0.53	0.42	0.27-0.68	0.82	0.70	0.40-1.05	0.51	0.43	0.32-0.67
Co	0.21	0.16	0.10-0.24	0.30	0.23	0.15-0.35	0.20	0.17	0.12-0.22
Cu	11.36	8.54	5.98-12.58	14.96	12.09	9.00-17.93	11.78	9.53	7.05-13.56
Pb	3.49	2.75	1.92-3.50	3.30	3.16	2.42-3.57	3.42	2.85	2.33-3.80
Mo	98.46	80.02	44.23-132.63	122.77	106.86	71.45-149.00	92.24	81.41	59.8-109.98
Ni	2.34	1.67	0.83-2.70	3.23	2.24	1.48-3.54	2.27	1.76	1.14-2.50
**Creatinine-corrected**									
As	21.72	20.57	16.05-24.38	24.02	22.52	18.18-26.14	22.87	21.23	18.18-24.53
Cd	0.49	0.43	0.31-0.61	0.48	0.42	0.32-0.61	0.48	0.44	0.31-0.61
Co	0.20	0.16	0.13-0.22	0.18	0.14	0.11-0.19	0.19	0.16	0.13-0.21
Cu	11.80	8.59	6.47-11.98	9.70	7.89	6.22-10.51	11.21	9.68	7.50-12.54
Pb	3.96	2.64	1.85-3.90	2.14	1.81	1.41-2.33	3.26	2.83	2.17-3.63
Mo	89.42	80.62	54.77-112.01	74.26	72.61	49.85-97.95	85.98	87.40	59.75-105.49
Ni	2.36	1.51	0.93-2.46	2.01	1.44	0.90-2.09	2.16	1.61	1.20-2.46
**UER**									
As	1.16	1.05	0.76-1.31	1.14	1.00	0.79-1.28	1.14	0.99	0.82-1.26
Cd	0.03	0.02	0.02-0.03	0.02	0.02	0.02-0.03	0.02	0.02	0.02-0.03
Co	0.01	0.01	0.006-0.01	0.01	0.01	0.005-0.01	0.01	0.01	0.006-0.01
Cu	0.63	0.45	0.32-0.61	0.44	0.35	0.28-0.49	0.54	0.45	0.37-0.59
Pb	0.21	0.13	0.09-0.21	0.10	0.08	0.06-0.11	0.16	0.13	0.10-0.18
Mo	4.73	3.97	2.63-6.10	3.45	3.26	2.31-4.41	4.21	3.99	3.22-5.25
Ni	0.12	0.08	0.04-0.13	0.10	0.07	0.04-0.10	0.11	0.08	0.06-0.11

Table S3. The variance apportionment of $\log_{10}$-transformed creatinine concentrations in the three sample types collected from 11 adults.

**Type of sample**	Total samples (*n*=529)	Days apart (*n*=326)	Months apart (*n*=265)
**Spot sample**			
ICC	0.12	0.09	0.15
Between persons σ^2 (%)[a]	0.008 (12%)	0.005 (9%)	0.011 (15%)
Within person, between day σ^2 (%)[b]	0.001 (2%)	0.001 (2%)	0.001 (1%)
Within person, within day σ^2 (%)[c]	0.058 (86%)	0.052 (89%)	0.063 (84%)
**First morning sample**[d]			
ICC	0.55	0.49	0.62
Between person σ^2 (%)[a]	0.021 (55%)	0.020 (49%)	0.027 (62%)
Within person σ^2 (%)[b]	0.017 (45%)	0.019 (51%)	0.015 (38%)
**24-h collection**[d]			
ICC	0.53	0.54	0.61
Between person σ^2 (%)[a]	0.009 (53%)	0.007 (54%)	0.014 (61%)
Within person σ^2 (%)[b]	0.008 (47%)	0.006 (46%)	0.009 (39%)

Days apart: days 0, 1, 2, 3, and 4; months apart: days 0, 30, 60, and 90. σ^2= variance. [a]The proportion of between-person variance to the total variance. [b]The proportion of within-person between-day variance to the total variance. [c]The proportion of within-person within-day variance to the total variance. [d]For first morning and 24-h urine collections, the distinction between within-day vs. between-day variability is not applicable with only one measurement per day.
